# Ecological stress memory in wood architecture of two Neotropical hickory species from central-eastern Mexico

**DOI:** 10.1186/s12870-024-05348-2

**Published:** 2024-07-06

**Authors:** Ernesto C. Rodríguez-Ramírez, Jonas Frei, Fressia N. Ames-Martínez, Anthony Guerra, Agustina R. Andrés-Hernández

**Affiliations:** 1 Laboratorio de Dendrocronología, Universidad Continental, Urbanización San Antonio, Avenida San Carlos 1980, Huancayo, Junín Peru; 2 Atelier foifacht, Juglandaceae expert, Schaffhausen, Switzerland; 3 Laboratorio de Biotecnología y Biología Molecular, Universidad Continental, Urbanización San Antonio, Huancayo, Peru; 4 Programa de Investigación de Ecología y Biodiversidad, Asociación ANDINUS, Calle Miguel Grau 370, Sicaya, Junín, Huancayo, Peru; 5 Programa de Pós-Graduação em Agronomia/Fisiologia Vegetal, Departamento de Biologia- Instituto de Ciências Naturais, Universidade Federal de Lavras, Lavras, Minas Gerais 7203-202 Brazil; 6 Facultad de Ciencias Biológicas, Benemérita Universidad Autónoma de Puebla, Puebla, Pue Mexico

**Keywords:** Drought stress, Resilience, Physiological ecology, Structure‒function relationship

## Abstract

**Background:**

Drought periods are major evolutionary triggers of wood anatomical adaptive variation in Lower Tropical Montane Cloud Forests tree species. We tested the influence of historical drought events on the effects of ecological stress memory on latewood width and xylem vessel traits in two relict hickory species (*Carya palmeri* and *Carya myristiciformis*) from central-eastern Mexico. We hypothesized that latewood width would decrease during historical drought years, establishing correlations between growth and water stress conditions, and that moisture deficit during past tree growth between successive drought events, would impact on wood anatomical features. We analyzed latewood anatomical traits that developed during historical drought and pre- and post-drought years in both species.

**Results:**

We found that repeated periods of hydric stress left climatic signatures for annual latewood growth and xylem vessel traits that are essential for hydric adaptation in tropical montane hickory species.

**Conclusions:**

Our results demonstrate the existence of cause‒effect relationships in wood anatomical architecture and highlight the ecological stress memory linked with historical drought events. Thus, combined time-series analysis of latewood width and xylem vessel traits is a powerful tool for understanding the ecological behavior of hickory species.

**Supplementary Information:**

The online version contains supplementary material available at 10.1186/s12870-024-05348-2.

## Background

The Tropical Montane Cloud Forest (TMCF; *sensu* Bruijnzeel et al. [1]) often exhibit a high diversity of relict-endemic biota, characterized by a small number of lineages adapted to wetter environments [2–4]. The decline in the tropical tree communities of key species (e.g., *Fagus*-*Magnolia*-*Quercus* forests, *Tilia*-*Acer*-*Quercus* forests, and *Carya*-*Quercus* forests) has been attributed to extreme climatic events [5–8]. Nonetheless, other factors, such as decreased precipitation, vapor plumes or recurring periods of hydric stress, have also been found to disrupt climatic fluctuations [9, 10]. During recurrent drought events, tree mortality is often caused by damage to the anatomical wood hydraulic supply network because of water stress [11, 12]. This ecophysiological mechanism has been identified as an ecological key [13, 14], that is linked to focusing on characteristics that define how organisms interact with their surrounding physical, chemical and biological environments [15, 16]; however, this relationship is not yet fully understood [17].

Recently, quantitative wood anatomy-climate link has received renewed attention because of its ecological signal of tree response to extreme climate events [16, 18, 19]. As extreme drought events become more frequent, plant communities have more robust mechanisms to cope with these damaging event [19, 20]. Nonetheless, current TMCF response to hydric stress may also bear the anatomical signatures of deep time ecological processes, driven by climate change [16, 21]. Among these mechanisms we find *ecological stress memory* (ESM, [5, 7]). The ESM retains a *stress imprint* as a plant’s ability to modify its epigenetic, physiological, and metabolomic processes which can affect plant resilience, recovery, tolerance, and resistance response [22–24].

Small moisture fluctuations can limit the prosperity of some TMCF tree species because these hydric deficit fluctuations lead to water stress, reducing growth and increased mortality of certain climate-sensitive tree species [25]. Three distinct types of TMCFs have been identified: Lower (LTMCF; >700–1700 m asl), Upper (UTMCF; 1701–1799 m asl), and Subalpine (STMCF; 1800–3500 m asl) [3, 26]. LTMCF and UTMCF are delimited by a persistent increase in cloud condensation, whereas the transition from STMCF occurs at temperatures ranging from 10 to 20 °C and with increased humidity and frequent cold and fog [27].

Although each TMCF type has its own predominant plant community, LTMCFs are most stressed by climate change and human activities such as logging, pastureland, cattle grazing, and corn crops [28]. Furthermore, a better long-term assessment of low vapor plumes seasons and anatomical responses to drought is required for relict LTMCF tree species (e.g., *Carya* spp., *Juglans* spp., *Tilia mexicana* Schltdl., and *Ulmus mexicana* (Liebm.) Planch.), given their isolated and fragmented distribution [29], as well as socioeconomic and ecological functions in eastern Mexican TMCFs [25].

In the LTMCF, tree species are simply one example of numerous hotspot areas worldwide, where the effects of climate change are felt in the complex ecological relationship between water and forests [30]. Climate projections indicate that the LTMCFs experienced a warming trend and an increase in the elevation of the 0 °C isotherm during the latter half of the 20th century [21, 31]. Furthermore, future climate predictions suggest that these vulnerable ecosystems will face lower overall water availability [32–34]. Drought makes TMCFs oversensitive to burning during logging [17, 35]. As a result, high uncertainty exists in current modeling efforts for the ESM of LTMCF tree species, especially when evaluating the precedent response of tree species to hydric stress.

The genus *Carya* Nutt. belongs to the ancient Juglandaceae family of deciduous angiosperms (late Cretaceous; ∼100.5–66 Ma; [36]) and shows phytogeographic disjunctions in eastern Asia and eastern North America [37]. In Mexico, the genus *Carya* (hickory) includes four species: *Carya illinoinensis* (Wangenh.) K. Koch, *C. ovata* var. *mexicana* (Engelm.) W.E. Manning, *C. myristiciformis* (F. Michx.) Nutt., and the endemic *C. palmeri* W.E. Manning (https://www.iucnredlist.org/search?query=Whickory&searchType=species) [37, 38], which show isolated distribution to LTMCFs of eastern Mexico. Nevertheless, it remains unclear how these trees may respond to such water stress events by adaptation to changes in climate through phenotypic plasticity, as their effects on the growth rings and xylem vascular characteristics of hickory species have not yet been investigated.

In this study, the following question was asked. Is ecological stress memory (ESM) recorded in wood anatomical traits of hickory species in lower Tropical Montane Cloud Forests? To answer this question, we assessed two hypotheses: (i) a decrease in latewood width during historical drought years, which could create lagged correlations between growth and historical hydric stress conditions; and (ii) past tree growth, in which hydric stress affects wood anatomical traits between successive drought events. The aims of our study were to:

i. Develop precisely dated tree ring chronologies for the two *Carya* species.
ii. Test the relationship between the latewood chronologies of *Carya* species and regional climate sensitivity.
iii. Exploring latewood width resistance, recovery, resilience, and decline between hickory species during historical droughts.
iv. Assess whether xylem vessel traits adjust similarly during historical hydric stress events.

## Results

### Growth-ring width chronology

**Fig. 1:**
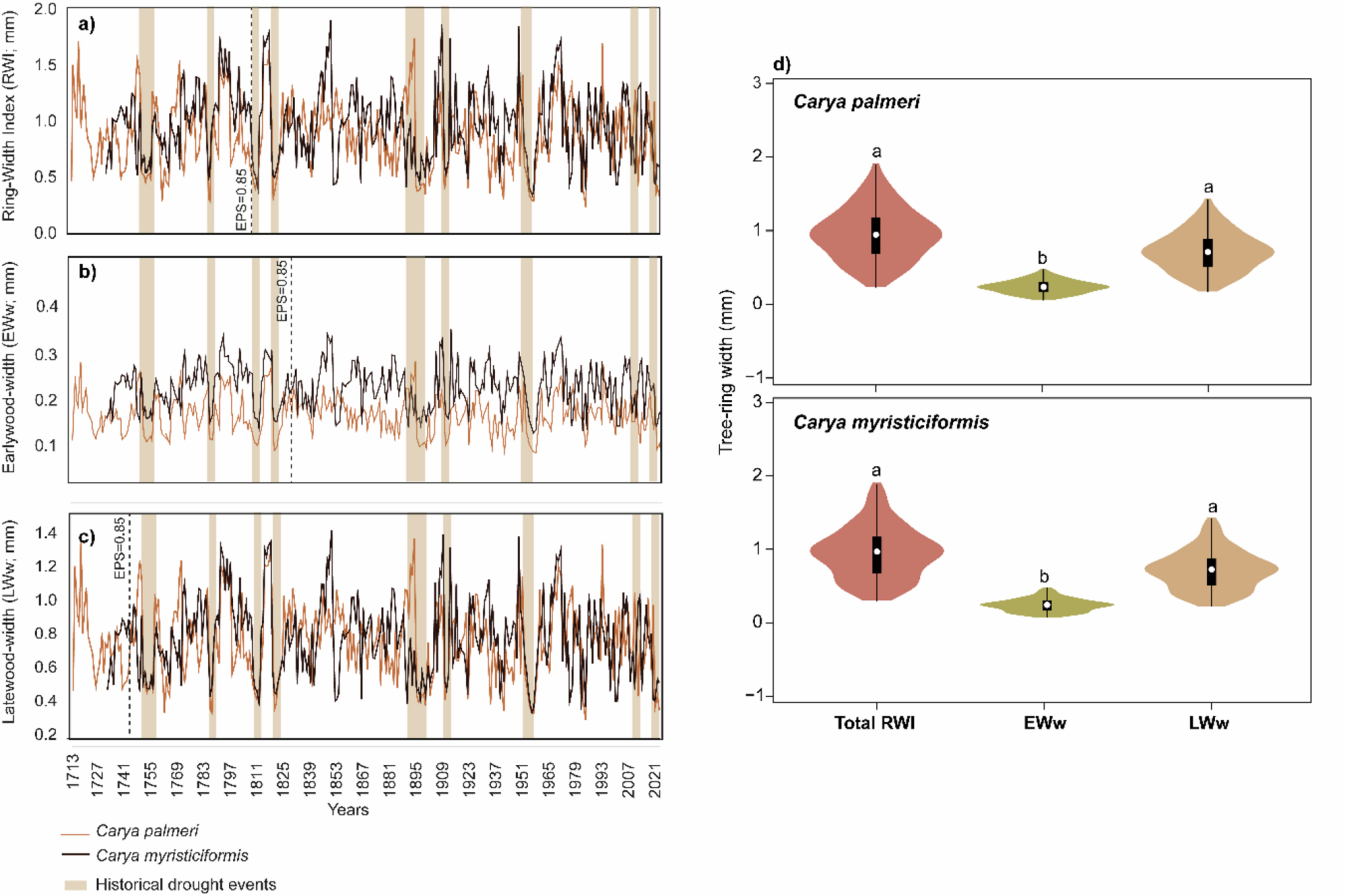
Ring-width chronologies: (**a**) Ring-Width Index (RWI), (**b**) Earlywood width (EWw), and (**c**) Latewood width (LWw) at the two hickory species (*Carya palmeri* and *C. myristiciformis*) and Mexican historical drought events [39, 40] from the Hickory Nut Forest of the “Los Planes” Private Conservation Area, located in a Lower Tropical Montane Cloud Forest in central-eastern Mexico. (**d**) Violin plots showing Ring-Width Index (RWI), Earlywood-width (EWw), and latewood-width (LWw) variation ranges between *Carya palmeri*; and *Carya myristiciformis*. Different letters indicate statistically significant differences (*p* < 0.05) using ANOVA and a *post-hoc* Tukey-Kramer HSD test

The independent hickory chronologies spanned 309 and 291 years for *Carya palmeri* and *C. myristiciformis*, respectively (Fig. 1a). A correlation between the two species was detected, where the mean sensitivity (MS) to climatic variables was high and similar between hickory species (Table 1). The $\stackrel{-}{R}$ values ranged from 0.40 (*C. myristiciformis*) to 0.45 (*C. palmeri*), and the EPS values ranged from 0.80 to 0.85, demonstrating that the RWI chronology exhibited good quality and a strong common signal among hickory trees. Nonetheless, the earlywood width (EWw; Fig. 1b) showed low MS, $\stackrel{-}{R}$, and EPS values, whereas the latewood width (LWw; Fig. 1c) displayed high MS, $\stackrel{-}{R}$, and EPS values between hickory species (Table 1). Finally, we detected specific tree growth depressions associated with historical drought (1750–1755, 1785–1786, 1808–1811, 1819–1823, 1894–1899, 1909–1910, 1951–1957, 2011–2012, and 2020–2022; Fig. 1).

Our analysis of variance (ANOVA) and *post-hoc* Tukey-Kramer HSD test revealed significant differences among RWI, EWw, and LWw. These differences may be influenced by specific climatic factors during specific growth periods, as shown in Fig. 1d. The RWI and LWw values were similar in hickory growth rings, which displayed high plasticity (ranging from 0.3 to 2 mm). Noteworthy, the EWw showed narrow values in both species (from 0.05 to 0.47 mm; Fig. 1d).

**Table 1:** Standard statistics for the RWI, EWw, and LWw chronologies of the two hickory species (*Carya Palmeri* and *Carya myristiciformis*)

Species	Chronology	Common period	Statistics		
			MS	EPS	$\stackrel{-}{R}$
*Carya palmeri*	RWI	1713–2022	0.28	0.85	0.45
	EWw	1720–2022	0.18	0.43	0.32
	LWw	1730–2022	0.43	0.96	0.56
*Carya myristiciformis*	RWI	1731–2022	0.31	0.80	0.40
	EWw	1760–2022	0.25	0.34	0.21
	LWw	1748–2022	0.38	0.90	0.60

MS: mean sensitivity, EPS: expressed population signal, $\stackrel{-}{R}$: mean correlation coefficient among tree-ring series

### Relationship between latewood width and climate

**Fig. 2:**
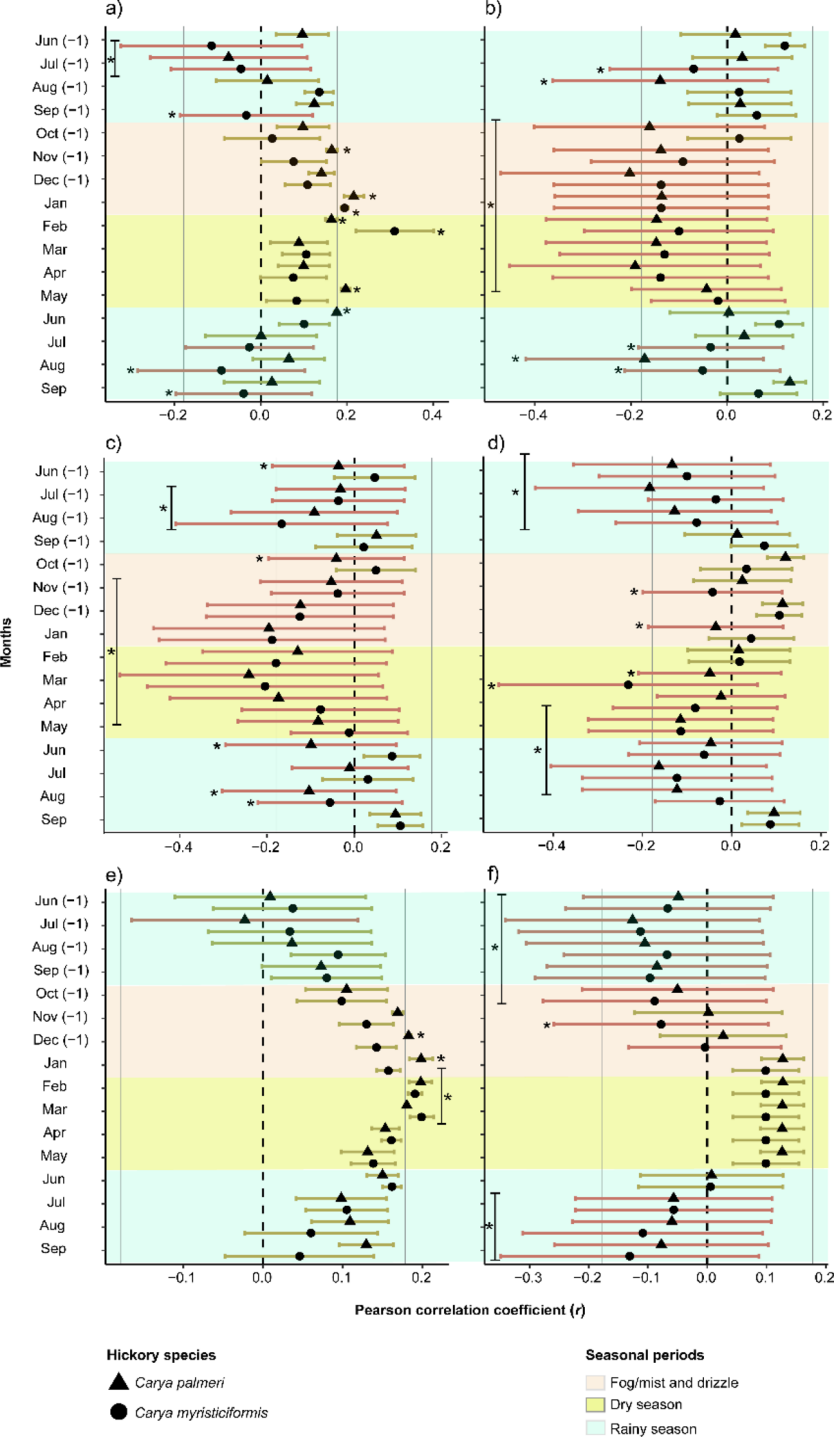
Climate-standardized latewood width correlations for all chronologies (using a 30-year spline with a 50% frequency cutoff after removing autocorrelation) with climatic variables for the period 1901–2022. Bars denote upper and lower confidence intervals (95%). (**a**) Monthly precipitation; (**b**) monthly evapotranspiration; (**c**) mean maximum temperature; (**d**) mean minimum temperature; (**e**) Palmer drought severity index; and (**f**) standardized precipitation-evapotranspiration index. *= Significant intervals (*p* < 0.05)

Our analysis revealed specific climatic cues that strongly influenced latewood-width growth (LWw) in both hickory species. The correlations between LWw and precipitation (Prec; Fig. 2a) indicated dissimilar negative effects (*r* ≤ −0.2) on *Carya palmeri* (July) and *C. myristiciformis* (from June to July and September) during the previous growing season. Furthermore, *C. myristiciformis* showed a significant negative effect in the current growing season from August to September. *C. palmeri*, in turn, exhibited a significant positive influence (*r* ≥ 0.2) on tree ring formation in November of the previous year, from January to February and from May to June, while *C. myristiciformis* showed a significant positive influence from January to February of the current growing season (Fig. 2a).

Evapotranspiration (EvT; Fig. 2b) showed consistent and negative responses (*r* ≤ −0.2 to −0.4) with both hickory chronologies from October to December of the previous growing season and from January to May of the current growing season. A noteworthy climatic cue was detected for *C. myristiciformis* during July (*r* = −0.30) of the previous growing season and from July to August of the current growing season, whereas *C. palmeri* exhibited a significant negative influence during August (*r* = −0.38) of the previous growing season and during August (*r* = −0.43) of the current growing season (Fig. 2b).

The mean maximum temperature (T_max_; Fig. 2c) triggered an evident negative effect from June to August in the previous growing season in both hickory chronologies (*r* ≤ −0.20 to −0.40); however, *C. myristiciformis* showed no effect during the previous June in the previous growing season. More specifically, T_max_ showed a negative influence on LWw growth rates in both species from October to December of the previous year and from January to June of the current growing season. Finally, the LWw for both hickory species was negatively correlated (*r* ≤ −0.20; Fig. 2c) between LWw and T_max_ beginning in the current August.

The mean minimum temperature (T_min_; Fig. 2d) showed consistent negative impact (*r* ≤ −0.20) on the two chronologies from June to August of the previous growing season. In contrast, *Carya myristiciformis* LWw was influenced during November of the previous growing season, while *C. palmeri* LWw was affected during January of the current growing season. Nonetheless, both also exhibited important negative correlations (*r* ≤ −0.2) in the current March. Albeit the species exhibited greater sensitivity from April to July in the current year of growth ring formation (Fig. 2d).

The PDSI (Fig. 2e) was significantly positively associated with the LWw of the hickory species (*r* ≥ 0.20). More specifically, *Carya palmeri* displayed positive correlations during the previous December and from January to March of the current growing season (*r* ≥ 0.20), whereas *C. myristiciformis* exhibited positive correlations from February to March (*r* > 0.20) (Fig. 2e).

Finally, for both hickory species, LWw was negatively correlated with the standardized precipitation-evapotranspiration index (SPEI6; Fig. 2f) from June to October of the previous growing season (*r* ≤ −0.20) of the two hickory species; likewise, there was a negative effect (*r* ≤ −0.20) on LWw from July to September of the current year of growth ring formation (Fig. 2f).

### Growth-ring indicators for detecting ecological stress tolerance

**Fig. 3:**
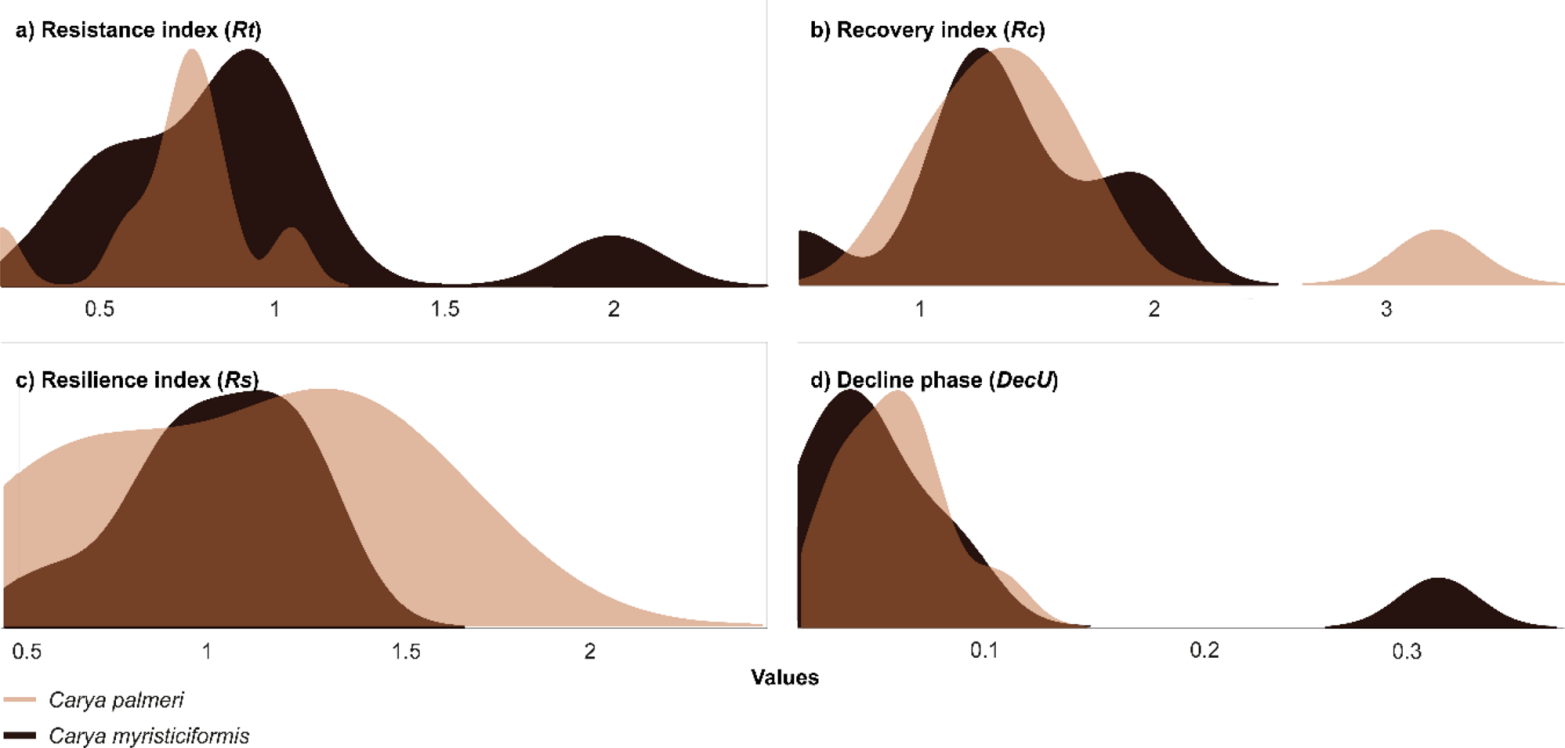
Kernel density plots demonstrating the LWw variation ranges in ecologically sensitive indicators (*Rt*, *Rc*, *Rs*, and *DecU*) between hickory species: (**a**) resistance index, (**b**) recovery index, (**c**) resilience index, and (**d**) decline phase

We analyzed the adaptability of two hickory species to hydric stress by comparing the development of LWw using Gaussian kernel density plots (Fig. 3). At first glance, the analysis revealed positive values; nevertheless, *Carya palmeri* showed narrow *Rt* values (*Rt* = 0.3–1.3; Fig. 3a), whereas *C. myristiciformis* revealed a high reversal capacity of the LWw in terms of ecological performance during drought periods (*Rt* = 0.3–2.5; Fig. 3a). Furthermore, both hickory species showed high recovery ability (*Rc*) relative to the damage incurred during periods of water stress (*Rc* = 1.50; Fig. 3b). Both hickory species showed a strong ability to increase their resilience (*Rs*) during frequent drought periods. The *Rs* values of *C. palmeri* ranged from 0.5 to 2, while those of *C. myristiciformis* ranged from 0.5 to 1.7 (Fig. 3c), indicating the occurrence of positive ecological stress. Finally, we observed low decline phase values (*DecU* ≤ 0.1) in the two hickory species during repeated stress events (Fig. 3d).

### Effect of climate on xylem vessel traits

**Fig. 4:**
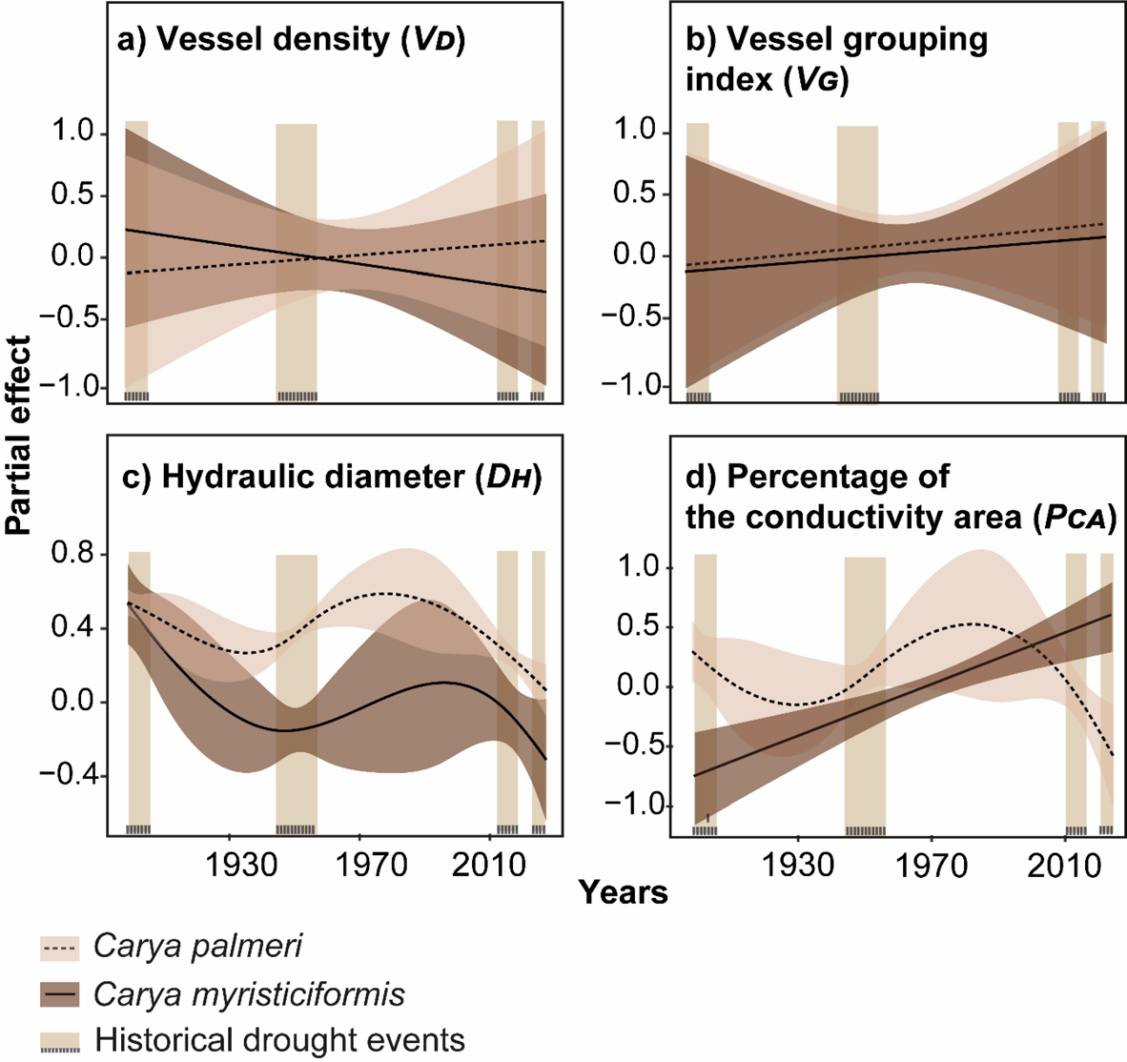
Generalized additive model (GAM) response curves depicting the relationships between the means of 4 wood anatomical trait variations per hickory species (*Carya palmeri* and *C. myristiciformis*) and climatic factors (P_rec_, EvT, T_max_, T_min_, PDSI, and SPEI6) were used as explanatory variables, whereas the following xylem vessel traits were considered response variables: (**a**) vessel density, (**b**) vessel grouping index, (**c**) hydraulic diameter, and (**d**) percentage of the conductivity area. The percentages of the total model deviance explained by each variable (Dev. %), REML values and adjusted R^2^ values associated with *p* values are listed in Table S2. Shadow bands represent 95% confidence intervals (1.96*SE) for the estimated wood anatomical trait variation based on GAM predictions. The vertical rectangles represent historical drought events (from 1901 to 2022)

The results of the GAM, ANOVA and BRT model (Boosted Regression Tree Model) indicated a significant difference (*p* < 0.05; Fig. 4) in wood anatomical traits between the climatic factors (P_rec_, EvT, T_max_, T_min_, PDSI, and SPEI6) during drought and non-drought periods (from 1901 to 2022). Summary of the GAMs between climatic factors and hickory species, ANOVA and *post-hoc* Tukey test results, and BRT model results and performance measures for drought and non-drought periods are summarized in the electronic supplementary material, Tables S2–S4. The *V*_*D*_ showed a significant response among the drought, predrought and post-drought periods of the two hickory species (Fig. 4a), even though EvT showed a positive trend (linear response) only in *Carya myristiciformis* (Fig. 4a). The two hickory species showed that the *V*_*G*_ exhibited a significant response during the drought and pre- and post-drought periods (Fig. 4b). *D*_*H*_ significantly responded to all climatic factors and to hydric stress and non-drought conditions in the two species (Fig. 4c). We observed a linear response with the *D*_*H*_, showing a positive trend with the T_max_, whereas with the EvT, the response exhibited a negative trend; nevertheless, both species exhibited a nonlinear response with the P_rec_, T_min_, PDSI, and SPEI6 (Fig. 4c). Finally, the *P*_*CA*_ revealed a significant response when the drought period ended, and a non-drought period began (Fig. 4d). Moreover, *Carya palmeri* showed a significant nonlinear response to T_max_ and SPEI6, whereas P_rec_ (lineal-negative trend) and T_min_ (nonlinear trend) demonstrated a significant response in *C. myristiciformis* (Fig. 4d).

We identified in the BRT model the performance results of each xylem vessel trait model that were above average for training data correlation (R^2^ > 0.50) and cross-validation data correlations (R^2^ > 0.50). The summary of BRT model results and performance measures for drought and non-drought periods are in the electronic supplementary material, Table S4). During the drought period, we observed that PDSI was affected to a greater percentage by *V*_*D*_ (29.81%) and *V*_*G*_ (49.71%), Prec to *D*_*H*_ (40.26%) and T_min_ to *P*_*CA*_ (27.29%) for *C. palmeri*. On the other hand, we observed that T_min_ affected *V*_*D*_ (37.75%), *V*_*G*_ (40.31%) and *D*_*H*_ (44.35%), finally, SPEI affected *P*_*CA*_ (73.14%) to *C. myristiciformis* (Table 2). In the non-drought period, T_min_ influenced on *V*_*D*_ (29.98%), EvT to *V*_*G*_ (60.92%), PDSI to *D*_*H*_ (24.75%) and T_max_ to *P*_*CA*_ (48.09%) for *C. palmeri*. Furthermore, Prec affected *V*_*D*_ (41.62%), PDSI to *V*_*G*_ (29.88%) and *D*_*H*_ (44.24%), finally T_max_ to *P*_*CA*_ (41.10%) for *C. myristiciformis* (Table 2).

**Table 2:** Importance and increment of node purity by BRT model

Functional traits	Climatic factors	*Carya palmeri*		*Carya myristiciformis*	
		%C-drought	%C-non drought	%C-drought	%C-non drought
*V*_*D*_	T_max_	11.41	18.55	14.18	5.28
	T_min_	23.38	29.98	37.75	23.56
	Prec	16.66	4.71	26.33	41.62
	EvT	3.29	6.88	4.87	4.71
	PDSI	29.81	24.93	5.46	23.10
	SPEI	15.46	14.93	11.41	1.81
*V*_*G*_	T_max_	32.61	5.88	16.51	19.49
	T_min_	1.87	17.45	40.31	7.52
	Prec	4.21	3.67	5.79	21.37
	EvT	7.78	60.92	11.31	15.70
	PDSI	49.71	11.3	12.25	29.88
	SPEI	3.82	0.74	13.83	6.02
*D*_*H*_	T_max_	9.14	15.22	14.37	19.48
	T_min_	9.25	10.10	44.35	4.67
	Prec	40.26	15.30	9.43	6.07
	EvT	2.87	14.81	11.64	14.74
	PDSI	32.35	24.75	8.45	44.24
	SPEI	6.13	19.82	11.75	10.79
*P*_*CA*_	T_max_	9.27	48.09	3.99	41.10
	T_min_	27.29	8.45	16.20	33.75
	Prec	11.76	5.97	0.70	15.17
	EvT	5.49	15.68	1.31	2.50
	PDSI	20.37	10.36	4.67	5.16
	SPEI	25.82	11.45	73.14	2.33

## Discussion

In this study, we demonstrated that the latewood anatomical traits of the two studied relict-hickory species signaled the impact of historical periods of water deficit through the anatomical adaptation of their growth width and xylem vessel [24, 41, 42], adjusting pre-drought event and post-drought across each drought event in the LTMCF of central-eastern Mexico. Nevertheless, the semi-ring-porous wood suggests moisture adaptations in both hickory species, and it is noteworthy that the latewood anatomy traits (i.e., latewood width, hydraulic diameter, and percentage of conductivity area) demonstrated a climatic imprint on ecological sensitivity indicators (resistance, recovery, resilience, and decline) to historical hydric stress because of their differences in adaptation potential of wood anatomy traits.

Our study showed that the anatomical adaptations of hickory wood are influenced by specific climatic cues. Overall, previous studies on other TMCF tree species, such as *Cedrela angustifolia* Sessé & Moc. ex DC., *Fagus mexicana*, *Juglans australis* Griseb., *J. neotropica* Diels, *Magnolia schiedeana* Schltdl., *M. vovidesii* A. Vázquez, Domínguez-Yescas & L. Carvajal, *Pinus pseudostrobus* var. *apulcensis* (Lindl.) Shaw, *Quercus delgadoana* S. Valencia, Nixon & L.M. Kelly, *Q. meavei* S. Valencia, Sabas & O.J. Soto, *Symplocos coccinea* Bonpl., and *S. speciosa* Hemsl. [16, 43–49], suggesting that moisture stress variations trigger anatomical adjustments in wood. We suggest that this wood anatomical adaptation could be used as a proxy to explore the influence of climate on anatomical adaptations in each TMCF type (e.g., UTMCF, LTMCF and UTMCF; [22]).

### Insight into long-term climatic effects on tree growth

Our findings showed that hickory species generated suitable growth rings for evaluating the radial growth rate (including early and late wood), age, adaptability, and forest climatic cue history. In this context, the present study provides relevant new data on the *ecological stress memory* (ESM) of hickory species in response to local historical drought events. Tree-ring data can provide insight into long-term tree growth trajectories after stress, and trees with slow post-stress recovery rates have significantly decreased adaptability [7, 23, 24], reflecting enhanced tree resistance, resilience, and/or recovery despite recurrent narrow tree-ring width–drought events after several decades [22]. The use of quantitative wood anatomy tools allowed us to assess the effects of historical drought on latewood width and xylem architecture dating back to 1713.

This study examined the correlation between the LWws of hickory species and specific climatic factors, including EvT, T_max_, T_min_, PDSI, and SPEI6, throughout the year. Notwithstanding, some species respond better to specific months because of to slightly different timing of growth in different species. Although EvT, T_max_, T_min_, PDSI, and SPEI6 have been use as tree growth estimators in the tropics [50, 51], there is no consensus on which index best explains the relationship between soil water availability and tree development. Even though canopy openness [52] or short dry periods over Mexican TMCFs could influence narrow ring formation during the midsummer short drought season (*canicula* period) [47]. These results may be attributed to the plant development period of the LTMCF hickory species, which is influenced by environmental oscillations such as low vapor plumes and drizzle-fog rates that impact the growing season [53, 54]. A similar climatic growth effect (summer temperatures from May to July) was detected in *Carya glabra* (Mill.) Sweet, *C. ovata* (Mill.) K. Koch, *C. alba* (L.) Nutt. [52, 55, 56], *C. cordiformis* (Wangenh.) K. Koch, *C. ovalis* (Wangenh.) Sarg., and *C. tomentosa* (Poir.) Nutt. in the Appalachian Mountains from the USA [57]. The results highlighted that hickory growth responses are consistent with the typical LTMCF climate influenced by warm-dry winters, daily temperatures > 13 °C, and summers with > 70% seasonal precipitation [1]. Variations in temperature (i.e., PDSI, T_max_, T_min_, and SPEI6) show a progressive decrease in moisture, such as in fog and mist [20, 58]; likewise, local LTMCF moisture variation may influence the growth sensitivity of hickory species [52]. In recent decades, these changes in precipitation patterns have been observed in other TMCFs worldwide [33, 59, 60].

### Effects of historical drought events on latewood growth

The sensitivity of LTMCF hickory species to historical drought events directly impacts capacity adaptation. Variations in rainfall patterns, including drizzle and fog, have decreased in parts of the tropics in recent decades [61] and are expected to continue and increase because of forest loss and global climate change [25, 31, 62].

By comparing the variations in resistance (*Rt*), recovery (*Rc*), resilience (*Rs*), and decline (*DecU*) indices along latewood width (LWw) during historical drought events, we found that transient climatic signals related to post-hydric stress and annual rings may contribute to improved tree adaptation to drought events over long timescales [13, 23, 63]. Both hickory species displayed a negative impact (*Rt* < 1) on the latewood width, indicating a strong influence of drought years. This is surprising given that both hickory species inhabit moist steep slopes. Previous studies have reported similar *Rt* values in response to hydric stress in other TMCF tree species, such as *Cedrela nebulosa* T.D. Penn. & Daza, and *Quercus meavei* [12, 16]. Despite, it is likely that the decrease in rainfall rates during the summer season, which is linked with increased temperatures, is the cause of this phenomenon [44] and/or depends in part on the consumption of stored reserves (i.e., hickory nut formation) during the stress event [64], resulting in a high ecophysiological impact (low resistance). Nevertheless, studies by Lloret et al. [22], Walter et al. [7], Mu et al. [24], and Brum et al. [14] have shown that individual adaptive fitness is influenced by recurrent stress episodes (e.g., fungal attacks, droughts, defoliation, and phenological processes) although and variability in response to different drought events [65]. Therefore, high *Rt* values could be directly composed of slow growth before drought events, preventing a significant decrease in development during drought periods [66].

Furthermore, hickory species exhibit high tolerance substandard to moisture, shade tolerance, and slow growth [52, 67], implying morphological adjust to prevent stress damage [7]. Both hickory species (*Carya palmeri* and *C. myristiciformis*) displayed low *Rc* values (with a mean *Rs* < 2), which could trigger reduced vigor linked to a decrease in tree growth [68] or reflect a survival strategy involving narrow tree-ring formation [63]. Nevertheless, low *Rc* values compensate for their growth reduction during drought, resulting in greater drought adaptation [69]. Notwithstanding, the *Rc* values were greater for trees that experienced a greater frequency of drought, as was the case for some *C. palmeri* and *C. myristiciformis* individuals. Rodríguez-Ramírez et al. [12] and Argüelles-Marrón et al. [16] also reported greater drought recovery in TMCF tree species (e.g., *Quercus meavei*, *Cedrela nebulosa*, and *C*. *angustifolia* DC.) with low growth rates. The relationship between structural and physiological adaptations, which are driven by regional climate, and the greater recovery of low-growth trees [62, 70] suggests that trees growing under more favorable conditions, such as moister climates and thus showing higher growth rates, may be less resistant and resilient to drought, even though they may recover faster [69].

The persistence of *Rs* in the two hickory species studied after low-growth episodes and the influence of predrought growth rates on the *Rt* and *Rc* indices suggest that autecological adaptations [71], microenvironmental features [72] or an ESM effect may be significantly influenced by the drivers of hydric stress events [7]. Alternatively, hickories can be highly resilient regardless of the impact of drought events. Although hickory species are relatively drought-tolerant, our results demonstrated decreased growth of *Rt* and *Rc* in response to extreme hydric stress periods at LTMCF. This is largely consistent with the patterns reported by Williams-Linera et al. [73] for Mexican TMCF tree species. In our case, this result likely reflects the higher frequency and severity of historical drought events occurring from 1750 to 2022 in the LTMCF studied. The impact of drought on tree-level *Rs* is not independent but rather dependent on how the trees were growing during the pre- and post-drought periods and on the type of site where they were growing [22]. Drought severity was found to significantly affect tree-level *Rs*, as reported by Bose et al. [74], Rodríguez-Ramírez et al. [12], and Argüelles-Marrón et al. [16]. The reported increases in forest mortality and low cloud cover associated with a changing climate, such as fog, drizzle, and vapor plumes [54], may be related to threshold effects on specific components of *Rs* values rather than an overall loss of resilience over time [22].

In hindsight, both hickory species exhibited low *DecU* values (< 0.2), indicating a slower decline rate [24], which reflects stronger resistance and slower recovery of hickories to hydric stress. Nonetheless, we showed that the hydraulic conductance of hickory trees decreased more slowly during the decline phase after they experienced stress, which resulted in a significant increase in their resistance to subsequent stress. Most likely, ESM acquired from precedent stress could promote plant *cross-stress memory* [75] because the plant coordinates its ecophysiological response memory at the cellular, organismal, and transcriptional levels to increase stress tolerance [14, 58].

### The architecture of xylem vessels relation to historical water stress: is there an ecological memory in the architecture of vessels?

Adaptations of xylem vessel architecture are important to understand how climate fluctuations directly impact hydraulic trade-off adaptations to stress [15, 76]. Drought events can imprint on xylem vessel traits in various ways. One of the most frequently measured anatomical traits associated with drought-induced embolism in angiosperms is vessel diameter [77]. Several studies have suggested that TMCF tree species adapt specific xylem vessel architectures to phenological (*Fagus mexicana*; [44]) and hydric stress periods (*Magnolia* spp., *Symplocos* spp., *Quercus* spp., *Cedrela* spp., and *Juglans neotropica*; [12, 16, 46–48]).

Xylem vessel traits have been found to be good predictors of climate variations, indicating their ecological role in the potential of trees to adapt to drought events [78–81]. We demonstrated that vessel-specific traits, i.e., *D*_*H*_ and *P*_*CA*_, are similarly adaptable across drought and pre- and post-drought events, indicating that climatic signals are key ecological components driving cross-drought tolerance [42]. These findings demonstrate the significant impact of drought events on xylem vessel traits; however, a single tree could hydraulically adjust under frequent hydric stress and potentially increase resilience [11]. This finding is supported by tree-ring evidence, which suggests that plants could imprint ESM through the adaptive ecophysiological ability to reshape their response to present stress based on past stress experiences [5, 7]. Nonetheless, it is necessary to evaluate how several TMCF tree species trigger autecological strategies in response to periodic stress events that enable them to be resilient to moisture stress periods [82].

## Conclusion

Anatomical and functional features of vessels and tracheids are important for understanding functional related to the environment [83, 84], although they are also essential for linking wood anatomy to ESM in TMCFs. The results presented here emphasize the sensitivity of TMCFs to hydric stress and suggest that current changes in hydrological processes, including fog, mist, vapor plumes, and drizzle [10, 54], will have direct consequences for drought resilience [17, 85].

## Materials and methods

### Study area and hickory species

The study was conducted in a Hickory Nut Forest of the “Los Planes” Private Conservation Area (20°25′ N, 98°77 W; total forest area: 74.72 ha), which is a Lower Tropical Montane Cloud Forest (LTMCF) in central-eastern Mexico (electronic supplementary material, Fig. 1a). The elevation ranged from 1411 to 1534 m asl. The climate is humid subtropical (Cwa *sensu* Peel et al. [86]) characterized by hot and humid summers, and cool to mild winters. The mean annual temperature is 18.9 °C, the mean annual precipitation is 947 mm and relative humidity is 70 − 90% (CLImate COMputing project; http://clicom-mex.cicese.mx/mapa.html; electronic supplementary material, Fig. 1a). The edaphic conditions of the Hickory Nut Forest investigated (electronic supplementary material, Fig. 1b) include Siltinovic soil (sj; [87]) from Cretaceous rock with sandy-silt-clay loam (pH from 4.5 to 6.5) [88].

*Carya palmeri* (common name: Mexican hickory, Squirrel Walnut, or Coamecate) (VU A2c; https://www.iucnredlist.org/species/66788384/66788386) and *Carya myristiciformis* (common name: Muskat-hickory) (LC; https://www.iucnredlist.org/species/62019640/62019642) (electronic supplementary material, Fig. 1c) are highly valued by humans (timber and walnuts) and are found in the fragmented LTMCF range of the Sierra Madre Oriental, central-eastern Mexico. Hickory tree species (up to 25 m tall) occur as scattered individuals or in small groups but rarely form pure stands [89]. In the Hickory Nut Forest studied, the species co-occurred with Petatillo (*Ulmus mexicana*), Nogal (*Juglans mollis* Engelm.), Cold jonote (*Tilia mexicana*) and several tropical oaks (*Quercus* spp.) [90]. Similarly, hickory species are associated with steep-slope ravines (≥ 20 °) and shaded valleys in the Northern Hemisphere (eastern Asia and eastern North America; [36]).

### Wood core sample collection

Thirty hickories (15 *Carya palmeri* and 15 *C. myristiciformis*) were sampled from the study forest (excluding trees with sores, rotting, and near grazing). Two wood cores were obtained from each tree with a 5 mm diameter Häglof^®^ increment borer (Långsele, Sweden) at 1.3 m above ground in two directions (parallel and perpendicular to the mountain slope; [91]). The wooden plugs used to fill the holes were sanitized with a mixture of 70% ethanol, 10% hydrogen peroxide, and 20% purified water [92]. A random selection of dominant hickory trees was made within the study forest to capture the widest range of environmental fluctuations, such as moisture and slopes [93].

The wood cores were air-dried, attached to wooden backings, and polished with a series of sandpapers of increasing coarseness (120, 180, 220, and 320 grit), followed by seven finer-grit sandpapers (400, 600, 800, 1000, 1500, 2000, and 2500 grit) [48]. A high-pressure vacuum was used to remove tyloses and wood dust from the interior of the conduits, allowing accurate xylem vessel identification [44].

### Tree-ring chronology development

The Ring-Width Index (RWI), earlywood-width (EWw) and latewood-width (LWw) of hickory species were measured separately to obtain intra-annual climate signals from the tree-ring parameters. Were used the presence of distinct narrow to wide earlywood conduits in a single intermittent row, and medium to narrow solitary and radial multiples of latewood conduits from two to three as phenological indicators to determine the EWw and LWw boundaries (https://www.wood-database.com). Growth rings (EWw and LWw) were measured under a stereoscopic microscope (Olympus SZ61, Olympus Corporation, Center Valley, PA, USA) and with a Velmex Tree Ring Measuring System (Velmex, Bloomfield, NY, USA) with 0.001 mm accuracy using TSAP-Win v. 4.67c [94]. The ring-width time series were visually and statistically cross-dated [95] using the software TSAP-Win and COFECHA [96]. To obtain the average of the detrended RWIs, EWw, and LWw, we standardized the raw ring-width series using the ARSTAN program [97] to remove non-climatic trends. A cubic smoothing spline with a 50% frequency cutoff at 30-year intervals was used to perform detrending on each series, which preserved high variance at a frequency equal to two-thirds of the length of each series [98]. The individual detrended tree-ring series were then averaged to build mean site chronologies by computing the bi-weighted robust mean [99]. To assess the reliability of the site chronologies, we used the expressed population signal (EPS > 0.85; [100]) and interseries correlation ($\stackrel{-}{R}$; [101]).

ANOVA and a *post-hoc* Tukey-Kramer HSD test was conducted to determine significant differences (*p* < 0.05) and compare the means of the RWI, EWw, and LWw values between hickory species. BoxPlotR (http://shiny-chemgrid.org/boxplot/; [102]) was used for the analyses.

### Climate growth relationships

We obtained climate data for monthly precipitation (P_rec_) and monthly evapotranspiration (EvT) in mm, mean maximum temperature (T_max_), mean minimum temperature (T_min_) in °C, the Palmer drought severity index (PDSI), and the standardized precipitation evapotranspiration index (SPEI6) from the CRU TS 4.0.3 dataset (resolution 0.5 ° intervals; https://climexp.knmi.nl/), with records dating from 1901 to 2020. The climate data used in this study were the average values of observational data over 10 years.

To assess the effect of local climatic signals from the previous growing season on latewood width (LWw, because it is more sensitive to climate than earlywood development; [103]), we performed Pearson’s correlation coefficient (*r*) using R software [104], which was computed between the LWw index series and monthly climatic data in a dendrochronological window, and calculated bootstrap response functions to test for significant correlations. We used the standard LWw chronology and monthly climate data for P_rec_, EvT, T_max_, T_min_, PDSI and SPEI6 for a period spanning from the previous growth year (June [− 1]) to the current growth year (September), and we used the ggplot2 package [105] for the graphics.

### (A) historical drought events

In Mexico, drought years from 1400 to 2022 were classified as severe, affecting economic production and people’s livelihoods [39]. Nonetheless, we selected severe drought events of 1750–1755, 1785–1786, 1808–1811, 1819–1823, 1894–1899, 1909–1910, 1951–1957, 2011–2012, and 2020–2022 [40]. Severe drought data were obtained from a network of 252 climate-sensitive tree-ring chronologies in and near Mexico [39]. We identified multi-year drought period with consistently low growth that deviate from long-term average [22, 106].

### Quantification of wood anatomical traits

To assess the influence of the ESM on wood anatomical traits, we identified the LWw formed during historical drought events (from 1901 to 2022) and for two consecutive years before and after historical drought events [44].

Digital wood cores were captured from each hickory species and saved in tiff format using a digital camera (Leica DFC 490) with a 10x objective and a resolution of 1.3 μm per pixel. The digital images were stitched using the software Adobe Illustrator CC v24.0.2 (www.adobe.com; [107]; electronic supplementary material, Fig. 1d). We considered four xylem vessel traits (vessel density, vessel grouping index, hydraulic diameter, and percentage of conductive area) related to various aspects of tree growth, xylem hydraulic conductivity, and overall performance, contributing to the ability of the species to adapt to different climatic factors. An overview interpretation of vessel anatomical traits, acronyms, measurements, and ecophysiological functions are summarized in the electronic supplementary material, table S1. All xylem vessel traits were calculated using ROXAS v3.0.560 software [108] and Image-Pro Plus v6.1 software (Media Cybernetics, Silver Spring, MD, USA).

### Drought stress signal on growth rings

The presence of ESM signals has been indicated by the observation of variations in ecological sensitivity indicators across multiple episodes of growth-induced stress [7, 20, 109]. To determine the effect of historical drought events on the LWw of each hickory species, we assessed four ecological sensitivity indicators, namely, resistance (*Rt*; Eq. 1), recovery (*Rc;* Eq. 2), resilience (*Rs;* Eq. 3), and decline (*DecU;* Eq. 4) [22, 24], in response to ecophysiological stress [33]. The indicators were evaluated at the individual tree level for each year between 1750 and 2022.

- The resistance (*Rt*) is estimated as the ratio between the ecological performance during and before disturbance. High resistance to disturbances reduces relative resilience, whereas low resistance increases it, and it is estimated as follows:

1 $$Rt=\frac{G{rowth \,ring}_{t}}{{Growth \,ring}_{t-2}}$$

(b) Recovery (*Rc*): the ability after a disturbance is estimated as the ratio between performance after and during the disturbance. Moderate stress can delay recovery trajectories and slow recovery rates. Severe stress can damage tissues and impair tree functioning. High post-recovery rates might reflect increased susceptibility to recurrent stresses. This value corresponds to the ratio between post-drought growth and growth during the drought period, and the following formula was used:

2 $$Rc=\frac{G{rowth \,ring}_{t+2}}{{Growth \,ring}_{t}}$$

(c) Resilience (*Rs*) is often estimated by analyzing the impact of disturbance on ecological properties. Values lower than 1 indicate that the effect of the event prevails after the disturbance. This ecological sensitivity indicator was calculated as follows:

3 $$Rs=\frac{G{rowth \,ring}_{t+2}}{{Growth \,ring}_{t-1}}$$

where the growth ring width of the annual growth in year *t* is represented by ‘growth ring_t_’. Growth ring_t−2_ represents the average ring width for the two years prior to year *t*, while Growth ring_t+2_ represents the average ring width for the two years following year *t*.

(d) Decrease (*DecU*): We identified the decline phase of each LWw as the period from the first year to the year of the latest minimum RWI of each narrow LWw influenced by drought events.

4 $$DecU=\frac{Pre5-Min}{Dt}$$

where *Pre5* is calculated by averaging the LWws 5 years before and after an LWw developed during a stress event (i.e., defoliation, drought, hurricanes and insect or fungal attack), which represent pre- and post-stress tree growth states, respectively. *Min* is the minimum RWI during a narrow RWI developed in a stress event. *Dt* is the number of years covered by the decline phase of a narrow RWI. Higher values of *DecU* indicate a faster rate of decrease, which reflects the weaker resistance of trees to stress.

To relate and explore the ecological sensitivity indicators (*Rt*, *Rc*, *Rs*, and *DecU*) between the hickory species LWw, we constructed Gaussian kernel density plots [110] and calculated skewness to describe the symmetry of the data distribution [111]. These analyses were performed in R software using the function *geom_density()* in the ggplot2 package [105].

### Ecological stress memory in xylem architecture adjustment

A generalized additive model (GAM; [112]) was used to assess the historical effects of climatic factors (P_rec_, EvT, T_max_, T_min_, PDSI, and SPEI6), including historical drought events (from 1901 to 2022), on xylem vessel traits (*V*_*D*_, *V*_*G*_, *D*_*H*_, *P*_*CA*_) between hickory species. The explanatory factors were climatic variables (fixed effects), whereas xylem vessel traits were response variables. To account for the potential lack of independence between sampled individuals and periods of drought and non-drought, we included individual trees and periods as random factors.

The significance of each explanatory factor was determined using the restricted maximum likelihood (REML; [113]). This score helps diagnose potential problems of under- or over-smoothing in mixed models. The optimal model for each scenario was then selected using the sample-corrected Akaike information criterion (AICc) [114]. The selection criterion for the best model was to identify cases where ΔAICc = 0, and analysis of variance (ANOVA) for model validation and Poisson distribution tests were performed using the QQ plots for each GAM. The analysis was performed using the mgcv [115] and aiccmosavg [116] R packages.

We performed a Boosted Regression Trees (BRT) method to improve the predictive performance of several single models [117]. We used four parameters: tree complexity (TC), learning rate (LR), bag fraction (BF) and number of trees (NT), which are regularized by setting and making a prediction [118] for the response variable in drought and non-drought periods. For each BRT model, we selected a combination of final values for LR, TC and BF, and number of trees based, which included maximizing the training data correlation, maximizing cross-validation correlation, and minimizing the residual deviation [118].

Finally, the optimal values of LR, TC and BF were set to 0.01, 5 and 0.5, respectively. This combination generates an optimal NT of at least ≤ 2500 trees using a 10-fold cross-validation method, to avoid overfitting the models [118]. We evaluated correlation (R^2^), Total Mean Deviance (TMD), Residual Mean Deviance (RMD) for trained data, and Estimated Deviance (ED) and Correl Partial dependence were also determined to assess the effect of explanatory factors for each response variable. All BRT analyses were performed using the R packages gbm [119] and dismo [120].

### Electronic supplementary material

Below is the link to the electronic supplementary material.

Supplementary Material 1

Supplementary Material 2

Supplementary Material 3

Supplementary Material 4

Supplementary Material 5

## Data Availability

All data generated or analysed during this study are included in this published article [and its supplementary information files].Chronologies have been deposited in the Dryad Digital Repository https://datadryad.org/stash/share/roEYX05QtE5uY_KVAkXj4pyBDbxFFy0FGNPM5xxZ4ng.
