# Supplementary material for: Ecological stress memory in wood architecture of two Neotropical hickory species from central-eastern Mexico
Source: BMC Plant Biol. 2024 Jul 6;24:638. doi: 10.1186/s12870-024-05348-2 (PMC11227188; doi:10.1186/s12870-024-05348-2)
Supplement: Supplementary file 1 — Supplementary Material 1 [file 12870_2024_5348_MOESM1_ESM.docx]

**Table S1**. Overview of vessel anatomical traits. Acronyms, measurements, and ecophysiological functions.

| **Vessel trait** | **Acronym** | **Measurement** | **Ecophysiological function** |
| --- | --- | --- | --- |
| Vessel density | *V_D_* | We selected the latewood area between two wood rays (an average of 7.8 mm width × 9.4 mm length) and calculated the number of vessels per square millimeter (mm^−2^). | Enhance hydraulic integration with track redundancy [114]. |
| Vessel grouping index | *V_G_* | $V_{G=}\frac{N conduits}{N grouping conduits}$ | Improve hydraulic integration and increase resilience to drought events [62]. |
| Hydraulic diameter | *D_H_* | $D_{H}\frac{\sum_{n=1}^{N} D_{n}^{5}}{\sum_{n=1}^{N} D_{n}^{4}}$ | Influencing hydraulic conductivity, adjustments, and tree size [106]. |
| Percentage of the Conductive Area | *P_CA_* | $P_{CA=}\frac{1}{\frac{x}{y}+1} x 100$ | Related to the efficient hydric and nutrients within the tree, which is essential for tree growth, development, and adaptation to changing environmental conditions [79]. |
