# Supplementary material for: Ecological stress memory in wood architecture of two Neotropical hickory species from central-eastern Mexico
Source: BMC Plant Biol. 2024 Jul 6;24:638. doi: 10.1186/s12870-024-05348-2 (PMC11227188; doi:10.1186/s12870-024-05348-2)
Supplement: Supplementary file 2 — Supplementary Material 2 [file 12870_2024_5348_MOESM2_ESM.docx]

**Table S2**. Summary of the GAMs between climatic factors and hickory species. var = climatic variable, edf = effective degrees of freedom, REML = restricted maximum likelihood, Dev. (%) = percent of deviance explained, and R^2^ = correlation coefficient adjusted. The numbers in bold indicate *p* < 0.05.

| **Wood anatomical traits** | **Explanatory variables** | | | | | | | | | | |
| --- | --- | --- | --- | --- | --- | --- | --- | --- | --- | --- | --- |
|  | Variables | *Carya palmeri* | | | | | *Carya myristiciformis* | | | | |
|  |  | edf | | REML | Dev. (%) | R^2^ | edf | | REML | Dev. (%) | R^2^ |
| *V_D_* | s(indiv) | 1.00 |  | 48.42 | 70.2 | 0.67 | 1.00 |  | 52.59 | 78.9 | 0.77 |
|  | s(P_rec_) | 1.0 |  |  |  |  | 1.00 |  |  |  |  |
|  | s(T_max_) | 1.00 |  |  |  |  | 1.00 |  |  |  |  |
|  | s(T_min_) | 1.00 |  |  |  |  | 1.00 |  |  |  |  |
|  | s(EvT) | 1.00 |  |  |  |  | **1.00** |  |  |  |  |
|  | s(PDSI) | 1.54 |  |  |  |  | 1.00 |  |  |  |  |
|  | s(SPEI6) | 1.00 |  |  |  |  | 1.00 |  |  |  |  |
|  | s(period) | **0.91** |  |  |  |  | **0.90** |  |  |  |  |
| *V_G_* | s(indiv) | 1.00 |  | 37.44 | 86.2 | 0.82 | 1.00 |  | 42.01 | 75.5 | 0.67 |
|  | s(P_rec_) | 1.00 |  |  |  |  | 1.00 |  |  |  |  |
|  | s(T_max_) | 1.00 |  |  |  |  | 1.00 |  |  |  |  |
|  | s(T_min_) | 1.00 |  |  |  |  | 1.00 |  |  |  |  |
|  | s(EvT) | 1.00 |  |  |  |  | 1.00 |  |  |  |  |
|  | s(PDSI) | 1.00 |  |  |  |  | 1.36 |  |  |  |  |
|  | s(SPEI6) | 1.00 |  |  |  |  | 1.00 |  |  |  |  |
|  | s(period) | **0.88** |  |  |  |  | **0.88** |  |  |  |  |
| *D_H_* | s(indiv) | 3.99 |  | 133.7 | 99 | 0.97 | 2.94 |  | 134.3 | 97.7 | 0.95 |
|  | s(P_rec_) | **3.39** |  |  |  |  | **4.76** |  |  |  |  |
|  | s(T_max_) | **1.00** |  |  |  |  | **1.00** |  |  |  |  |
|  | s(T_min_) | **5.15** |  |  |  |  | **2.92** |  |  |  |  |
|  | s(EvT) | **1.00** |  |  |  |  | **1.00** |  |  |  |  |
|  | s(PDSI) | **1.43** |  |  |  |  | **2.72** |  |  |  |  |
|  | s(SPEI6) | **4.98** |  |  |  |  | **3.48** |  |  |  |  |
|  | s(period) | **2.99** |  |  |  |  | **0.99** |  |  |  |  |
| *P_CA_* | s(indiv) | 3.34 |  | 87.59 | 95.6 | 0.93 | 1.00 |  | 95.36 | 92.1 | 0.91 |
|  | s(Prec) | 1.00 |  |  |  |  | **1.00** |  |  |  |  |
|  | s(T_max_) | **3.28** |  |  |  |  | 1.00 |  |  |  |  |
|  | s(T_min_) | 2.32 |  |  |  |  | **6.02** |  |  |  |  |
|  | s(EvT) | 1.00 |  |  |  |  | 2.15 |  |  |  |  |
|  | s(PDSI) | 1.00 |  |  |  |  | 1.57 |  |  |  |  |
|  | s(SPEI6) | **3.26** |  |  |  |  | 1.00 |  |  |  |  |
|  | s(period) | **2.96** |  |  |  |  | **0.96** |  |  |  |  |

s= smoothing in the mixed models
