# Supplementary material for: Ecological stress memory in wood architecture of two Neotropical hickory species from central-eastern Mexico
Source: BMC Plant Biol. 2024 Jul 6;24:638. doi: 10.1186/s12870-024-05348-2 (PMC11227188; doi:10.1186/s12870-024-05348-2)
Supplement: Supplementary file 3 — Supplementary Material 3 [file 12870_2024_5348_MOESM3_ESM.docx]

**Table S3**. Summary of the one-way ANOVAs measurements performed to test the effects of the *Carya* species, and drought vs. non-drought periods and their interaction, on four xylem vessel traits measured in hickory species. Response variables: *V_G_*, vessel grouping; *V_D_*, vessel density; *D_H_*, hydraulic diameter; *P_CA_*, Percentage of Conductivity Area. ** = *p* <0.05, *** = *p* <0.01

Vessel density (*V_D_*)

|  | Df | Sum Sq | Mean Sq | F value | Pr(>F) |  |
| --- | --- | --- | --- | --- | --- | --- |
| Drought | 1 | 63.40 | 63.40 | 740.607 | <2e-16 | *** |
| Non-drought | 1 | 5.85 | 5.85 | 68.341 | 7.64e-16 | *** |
| Drought:Non-drought | 1 | 0.10 | 0.10 | 1.159 | 0.282 |  |
| Residuals | 620 | 56.16 | 0.09 |  |  |  |

Differences Tukey: b a d c

Vessel grouping index (*V_G_*)

|  | Df | Sum Sq | Mean Sq | F value | Pr(>F) |  |
| --- | --- | --- | --- | --- | --- | --- |
| Drought | 1 | 99.69 | 99.69 | 818.95 | <2e-16 | *** |
| Non-drought | 1 | 110.11 | 110.11 | 904.61 | <2e-16 | *** |
| Drought:Non-drought | 1 | 3.26 | 3.26 | 26.75 | 3.04e-07 | *** |
| Residuals | 620 | 83.50 | 0.12 |  |  |  |

Differences Tukey: b a d c

Hydraulic diameter (*D_H_*)

|  | Df | Sum Sq | Mean Sq | F value | Pr(>F) |  |
| --- | --- | --- | --- | --- | --- | --- |
| Drought | 1 | 98.85 | 98.85 | 448.02 | <2e-16 | *** |
| Non-drought | 1 | 24.46 | 24.46 | 110.85 | <2e-16 | *** |
| Drought:Non-drought | 1 | 1.71 | 1.71 | 7.73 | 0.00559 | ** |
| Residuals | 620 | 144.73 | 0.22 |  |  |  |

Differences Tukey: a ab d c

Percentage of conductivity area (*P_CA_*)

|  | Df | Sum Sq | Mean Sq | F value | Pr(>F) |  |
| --- | --- | --- | --- | --- | --- | --- |
| Drought | 1 | 32.69 | 32.69 | 201.625 | <2e-16 | *** |
| Non-drought | 1 | 38.30 | 38.30 | 236.228 | <2e-16 | *** |
| Drought:Non-drought | 1 | 0.02 | 0.02 | 0.148 | 0.7 |  |
| Residuals | 620 | 111.22 | 0.16 |  |  |  |

Differences Tukey: d bc b a
