# Supplementary material for: Ecological stress memory in wood architecture of two Neotropical hickory species from central-eastern Mexico
Source: BMC Plant Biol. 2024 Jul 6;24:638. doi: 10.1186/s12870-024-05348-2 (PMC11227188; doi:10.1186/s12870-024-05348-2)
Supplement: Supplementary file 4 — Supplementary Material 4 [file 12870_2024_5348_MOESM4_ESM.docx]

**Table S4.** Summary of Boosted Regression Tree Model Results and performance measures for drought and non-drought periods of two hickory species of vessel density (*V_D_*), vessel grouping index (*V_G_*), hydraulic diameter (*D_H_*), and percentage of the conductivity area (*P_CA_*). R^2^= factor of correlation, TMV= Total Mean Deviance, RMV = Residual Mean Deviance, NT = Number of trees, ED = Estimated Deviance.

|  | Model Response Variable | Period | Trained Data | | | | Cross-Validated Data | |
| --- | --- | --- | --- | --- | --- | --- | --- | --- |
|  |  |  | R^2^ | TMV | RMV | NT | R^2^ | ED |
| *Carya palmeri* | *V_D_* | Drought | 0.87 | 0.960 | 0.697 | 2,500 | 0.79 ± 0.10 | 0.58 ± 0.18 |
|  |  | Non-drought | 0.74 | 1.026 | 0.688 | 2,150 | 0.83 ± 0.06 | 0.58 ± 0.11 |
|  | *V_G_* | Drought | 0.78 | 0.649 | 0.336 | 1.850 | 0.77 ± 0.13 | 0.37 ± 0.19 |
|  |  | Non-drought | 0.69 | 0.204 | 0.127 | 2,050 | 0.83 ± 0.15 | 0.37 ± 0.19 |
|  | D_H_ | Drought | 0.83 | 331.76 | 199.2 | 2,400 | 0.64 ± 0.05 | 0.11 ± 0.03 |
|  |  | Non-drought | 0.72 | 890.4 | 615.6 | 2,300 | 0.60 ± 0.07 | 0.11 ± 0.02 |
|  | *P_CA_* | Drought | 0.76 | 14.37 | 11.54 | 2,500 | 0.61 ± 0.06 | 0.41 ± 0.15 |
|  |  | No-drought | 0.73 | 73.96 | 46.03 | 2,500 | 0.71 ± 0.04 | 0.41 ± 0.15 |
| *Carya myristiciformis* | *V_D_* | Drought | 0.74 | 1.449 | 1.019 | 2,500 | 0.76± 0.10 | 0.53 ± 0.18 |
|  |  | No-drought | 0.69 | 1.597 | 0.878 | 2,250 | 0.79 ± 0.07 | 0.53 ± 0.21 |
|  | *V_G_* | Drought | 0.69 | 0.907 | 0.490 | 2,000 | 0.78 ± 0.14 | 0.45 ± 0.22 |
|  |  | No-drought | 0.58 | 0.372 | 0.253 | 2,350 | 0.83 ± 0.16 | 0.47 ± 0.16 |
|  | *D_H_* | Drought | 0.73 | 277.1 | 176.6 | 2,500 | 0.63 ± 0.07 | 0.12 ± 0.01 |
|  |  | No-drought | 0.70 | 912.9 | 599.3 | 2,350 | 0.68 ± 0.09 | 0.14 ± 0.03 |
|  | *P_CA_* | Drought | 0.78 | 18.65 | 9.171 | 1,900 | 0.62 ± 0.07 | 0.19 ± 0.03 |
|  |  | No-drought | 0.75 | 138.5 | 88.48 | 2,350 | 0.67 ± 0.11 | 0.19 ± 0.02 |
