# Supplementary material for: Ecological stress memory in wood architecture of two Neotropical hickory species from central-eastern Mexico
Source: BMC Plant Biol. 2024 Jul 6;24:638. doi: 10.1186/s12870-024-05348-2 (PMC11227188; doi:10.1186/s12870-024-05348-2)

**Figure S1**. a) Map showing the location of the Hickory Nut Forest of the “Los Planes” Private Conservation Area, which is located in a Lower Tropical Montane Cloud Forest in central-eastern Mexico, and Walter climatic diagrams of Los Planes, San Bartolo Tutotepec, Hidalgo (1980–2018; 20°25′ N, 98°77 W; 1550 m asl); blue-filled tips indicate high moisture. b) View of the studied Hickory Nut Forest. c) Nuts of *Carya palmeri* and *Carya myristiciformis*. d) Schematic representation of Carya digital cross-section images

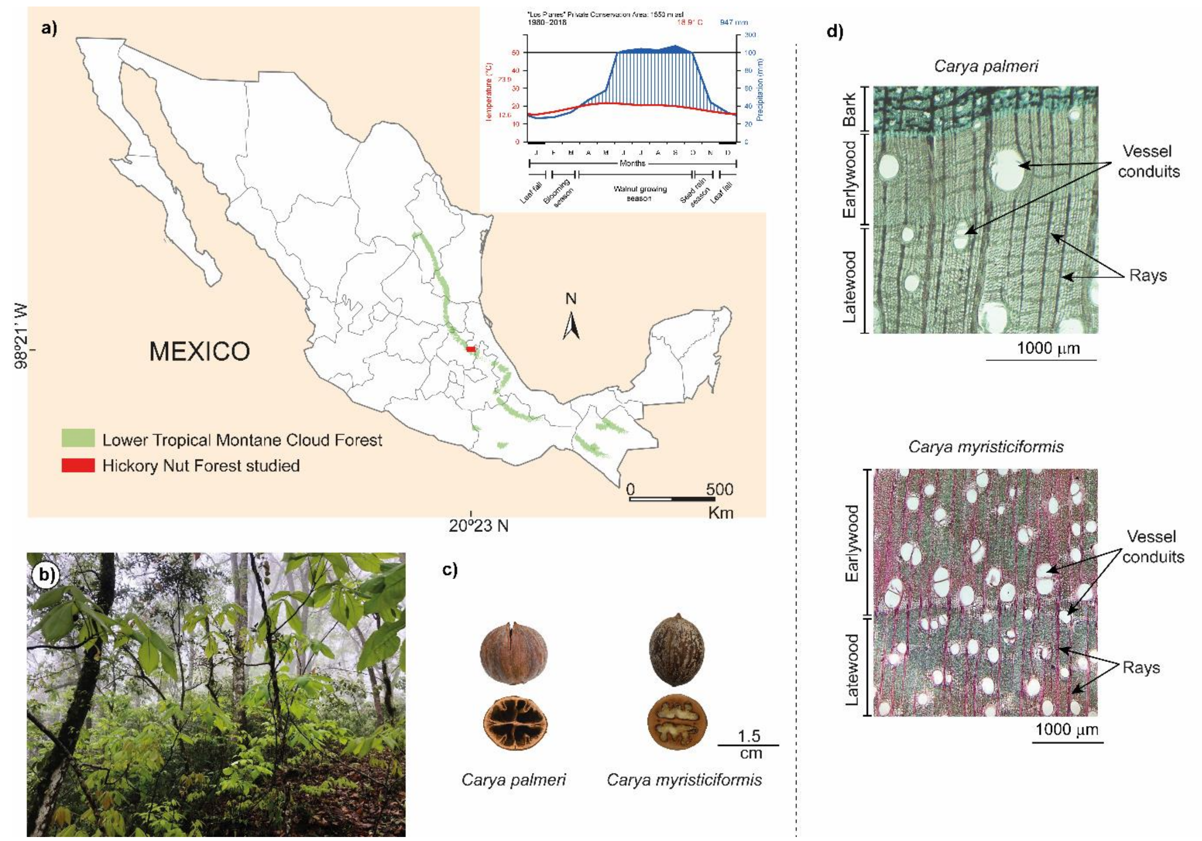

Supplement: Supplementary file 5 — Supplementary Material 5 [file 12870_2024_5348_MOESM5_ESM.pdf]
